# Impact of CAD-CAM technology and abutment angulation on prosthesis design for anterior maxillary implants: An original research study

**DOI:** 10.6026/973206300211662

**Published:** 2025-06-30

**Authors:** V. Santoshi Kumari, Kasi Reddy Snehalatha Reddy, Anusha Borra, Vattigunta Sravani, Nirban Mitra, Gadusu Bharath Kumar Reddy

**Affiliations:** 1 Department of Prosthodontics, Malla Reddy institute of dental Sciences, Malla Reddy Vishwavidyapeeth, Hyderabad, India; 2 Department of Prosthodontics, G. Pulla Reddy Dental College And Hospital, Kurnol, Andhra Pradesh, India

**Keywords:** Anterior maxillary implants, CAD-CAM technology, abutment angulation, prosthetic success, esthetic outcomes, patient satisfaction, layered zirconia, monolithic zirconia, lithium disilicate, canine-guided occlusion

## Abstract

The clinical performance of anterior maxillary implants restored using CAD-CAM technology, emphasizing the influence of abutment angulation, restorative material and occlusal scheme on treatment outcomes is of interest. Among 49 patients assessed, implants with 0° abutments exhibited superior prosthetic success, marked by minimal complications and enhanced retention. Layered zirconia restorations delivered the most favorable esthetic results, while canine-guided occlusion correlated with heightened patient satisfaction. Thus, the pivotal role of precise angulation, material selection and occlusal dynamics in optimizing the functional and aesthetic success of anterior implant restorations is shown.

## Background:

Dental implantology has undergone significant advancements over the past few decades, particularly with the integration of computer-aided design and manufacturing (CAD-CAM) technology. This technology has revolutionized prosthetic dentistry, enabling highly precise and customized implant restorations, thus improving the outcomes of implant-supported prostheses [1, 2]. The application of CAD-CAM technology allows for the fabrication of custom abutments, crowns and other prosthetic components with enhanced accuracy and fit [3]. Furthermore, the angulation of the abutment, which determines the alignment of the prosthesis relative to the implant axis, plays a crucial role in the functional and esthetic success of the restoration [4]. Historically, dental implants have evolved from rudimentary designs to highly sophisticated systems that offer greater predictability in both placement and restoration. Early implant systems, such as those developed by Brånemark in the 1960s, laid the foundation for modern implantology [5]. However, the challenge of achieving optimal esthetics and function, particularly in the anterior maxilla, has remained. The advent of CAD-CAM technology has addressed some of these challenges by enabling better customization of the prosthetic components to the individual's anatomical and functional needs [6, 7]. One of the most debated aspects of implant restoration is the angulation of the abutment. The angulation affects not only the esthetic outcome but also the retention and long-term stability of the prosthesis. Studies have suggested that abutment angulation can influence complications such as screw loosening and fractures, with some studies showing that more angled abutments may result in higher complication rates (Misch, 2015) [8]. On the other hand, abutments with minimal angulation tend to offer better fit and function but may not be ideal in cases where implant placement is not optimal or when achieving the desired esthetics requires angulation adjustments [9]. The materials used in implant restorations; including zirconia, lithium disilicate and porcelain, also significantly impact the esthetic outcomes and functional longevity of the prosthesis. Zirconia, both in its monolithic and layered forms, has gained popularity due to its strength and natural appearance. Lithium disilicate, known for its translucency and lifelike esthetics, is often used in anterior regions where appearance is critical (Sailer *et al.* 2007) [10].

This study aims to evaluate the influence of CAD-CAM technology and prosthetic abutment angulation on the clinical outcomes of anterior maxillary implant restorations. By analyzing the relationship between abutment angulation, material selection and patient satisfaction, this research will provide valuable insights into how these factors contribute to the success of implant-supported prostheses. Additionally, the study will explore the role of occlusal schemes (canine-guided vs. group function) in optimizing functional outcomes and patient comfort. The findings from this study will help refine treatment planning strategies and provide a better understanding of how to achieve superior esthetic and functional results in anterior implant restorations. Numerous studies have explored the impact of abutment angulation on implant restoration outcomes. A study highlighted that angulation plays a crucial role in both the biomechanical performance and the esthetic integration of the prosthesis [11]. Specifically, they found that while higher angulations (15° or more) could lead to esthetic improvements in cases where implants were placed with less ideal trajectories, they also increased the risk of complications like screw loosening and fractures (Misch, 2015) [8]. In terms of material selection, zirconia has long been favored in implant prosthetics for its strength and esthetics. Studies by Sailer *et al.* (2007) [10] demonstrated that monolithic zirconia restorations, although highly durable, lacked the translucency of other materials, which can be a disadvantage in the anterior region where esthetics are paramount. Layered zirconia and lithium disilicate have been shown to provide superior esthetic results due to their lifelike translucency and color-matching capabilities [12]. Additionally, the occlusal scheme plays an essential role in ensuring the long-term stability and function of implant-supported prostheses. Canine-guided occlusion has been advocated for its ability to reduce stress on the prosthesis, while group function occlusion has been shown to offer more versatility in patients with varying bite patterns [13]. Therefore, it is of interest to describe the Impact of CAD-CAM technology and abutment angulation on prosthesis design for anterior maxillary implants.

## Materials and Methods:

This prospective clinical study aimed to evaluate the impact of CAD-CAM technology and prosthetic abutment angulation on the design, material selection and clinical outcomes of anterior maxillary implant prostheses. A total of 49 patients, aged between 25 and 60 years, were included based on predefined inclusion and exclusion criteria, ensuring a homogeneous representation of prosthetic configurations and angulations. The patients required single or multiple anterior maxillary implants in the regions of central incisors, lateral incisors, or canines. The inclusion criteria required that patients be in good oral health, have adequate bone volume for implant placement and be capable of adhering to post-operative care instructions. Exclusion criteria included patients with uncontrolled systemic diseases, smokers who consumed more than 10 cigarettes per day, individuals requiring bone grafting due to insufficient bone volume and those with a history of previous dental implant failure in the anterior maxilla. The implants used in the study were from the Nobel Biocare Active^TM^ or Straumann® BLX systems, with lengths ranging from 10 to 13 mm and diameters between 3.3 and 4.1 mm. Delayed implant placement with immediate provisionalization was used in 30 cases. The prosthetic abutment angulation was classified into three groups: 0° (Straight) with 16 samples, 15° Angled with 18 samples and 25° Angled with 15 samples. Custom abutments were designed using advanced CAD software (Exocad or 3Shape Dental System), ensuring the abutments' angulation was tailored to the specific anatomical and implant trajectory requirements of each patient (Table 1 - see PDF).

To ensure precision in the fabrication of the prostheses, CAD-CAM technology was utilized throughout the study. Intraoral scanning was carried out using the 3 Shape TRIOS scanner, which provided detailed digital impressions of the implant sites. Prostheses were then designed using the Exocad or 3Shape Dental System software and fabricated with the Amann Girrbach Ceramill milling machine, ensuring high-quality fit and function. The materials used for prosthesis fabrication were selected to optimize both esthetics and durability. These materials included monolithic zirconia (n = 25 implants), layered zirconia with porcelain veneer (n = 10 implants) and lithium disilicate (E.max) (n = 14 implants). Prostheses were designed based on the principles of functional and esthetic excellence, with customized emergence profiles for each patient. The crowns were categorized as screw-retained (35 cases) or cement-retained (14 cases), depending on clinical judgment and individual patient factors. Gingival contours were evaluated using soft tissue simulation software to ensure the prostheses integrated seamlessly with the surrounding tissues. The occlusal scheme was tailored to the needs of each patient, with 30 patients receiving a canine-guided occlusion and 19 patients receiving a group function occlusion. All prostheses were designed with anterior guidance, minimizing posterior interference to avoid occlusal disharmony. Data were analyzed using SPSS v25 software. Descriptive statistics were used to summarize demographic and clinical characteristics. Comparative analysis was conducted using Chi-square tests to examine the relationship between abutment angulation and prosthetic complications, such as screw loosening and fractures. ANOVA was utilized to compare the outcomes between different occlusal schemes (canine-guided vs. group function). A significance level of p < 0.05 was considered statistically significant. The primary outcome measures included prosthetic success, assessed by clinical outcomes such as retention, screw loosening and fracture rates. Esthetic ratings were obtained through a Visual Analog Scale (VAS), with scores provided by a panel of dental professionals evaluating the appearance and integration of the prosthesis. Patient satisfaction was assessed using a post-treatment questionnaire that focused on comfort, esthetic satisfaction and overall functional outcomes of the implant prostheses.

## Results:

The study evaluated 49 patients who underwent anterior maxillary implant placement using CAD-CAM technology, with varying abutment angulations. The clinical outcomes, including prosthetic success, esthetic ratings and patient satisfaction, were analyzed based on abutment angulation, occlusal scheme and material selection.

## Prosthetic success:

The prosthetic success was evaluated by assessing retention, screw loosening and fracture rates. The results were categorized based on abutment angulation and a comparison was made across the three groups (0°, 15° and 25°). The rates of complications were recorded and analysed (Table 2 - see PDF). The analysis revealed a significant difference in screw loosening between the 0° and 15° abutment groups, with the 15° angled group having a higher complication rate. The fracture rate also varied, with a higher incidence in the 25° angulation group compared to the 0° and 15° groups (p = 0.035).

## Esthetic rating:

The esthetic outcome of the prostheses was evaluated using a Visual Analog Scale (VAS), which was rated by a panel of dental professionals. The results were analyzed for each abutment angulation and material used (Table 3 - see PDF). The results indicated that the esthetic outcomes were significantly better for layered zirconia compared to monolithic zirconia and lithium disilicate (p = 0.004). Furthermore, the 0° angulation showed higher VAS scores compared to the 15° and 25° angled abutments.

## Patient satisfaction:

Patient satisfaction was assessed using a post-treatment questionnaire. The responses were categorized based on comfort, esthetic satisfaction and overall functional outcomes. The analysis of variance (ANOVA) was performed to compare the satisfaction scores across different groups (Table 4 - see PDF). The overall patient satisfaction was highest for the 0° abutment angulation group, with a significant difference observed when compared to the 25° angulation group (p = 0.012). Comfort and esthetic satisfaction were also rated higher for the 0° abutment angulation.

## Occlusal scheme:

The two occlusal schemes-canine-guided and group function-were compared to assess their influence on functional outcomes and patient satisfaction (Table 5 - see PDF). The results demonstrated a statistically significant higher patient satisfaction in the canine-guided occlusion group, with better comfort, esthetics and overall functional outcomes (p = 0.045). For all the analyses, statistical significance was determined at the p-value threshold of <0.05. The results reveal that both abutment angulation and occlusal scheme significantly affect prosthetic success, esthetic outcomes and patient satisfaction. The differences in the complication rates, esthetic ratings and patient satisfaction scores were significant between the groups, as outlined in the respective tables.

## Discussion:

The results of this study evaluated the clinical outcomes of anterior maxillary implants with varying abutment angulations (0°, 15° and 25°) and assessed their effects on prosthetic success, esthetic ratings and patient satisfaction. The findings reveal significant differences in the complication rates, esthetic outcomes and patient satisfaction between the groups, providing important insights into how abutment angulation, material selection and occlusal scheme can impact the success of dental implants. Dental implants have revolutionized restorative dentistry, providing a reliable solution for tooth replacement. However, implant success is influenced by several factors, including implant placement, abutment angulation, material selection and occlusal scheme. Over the years, numerous studies have explored these variables to optimize implant outcomes. While some studies suggest that straight abutments (0°) yield superior results in terms of esthetics and function (Misch, 2015) [8], others propose that angulated abutments (15° and 25°) offer more flexibility in terms of implant positioning, particularly when dealing with limited space or poor bone quality (Albakri 2024) [14]. However, the potential for complications such as screw loosening, fractures and poor esthetic outcomes associated with increased angulation has led to concerns about the clinical implications of using angulated abutments. Understanding these implications is crucial, as implant-based rehabilitations are becoming increasingly common in both esthetic and functional treatments.

## Prosthetic success:

In terms of prosthetic success, screw loosening and fracture rates were used as indicators of complications. The study demonstrated that the 15° abutment angulation group had a significantly higher rate of screw loosening compared to the 0° group (p = 0.035). Additionally, the 25° angulation group had a higher fracture rate compared to both the 0° and 15° groups. These findings are consistent with previous studies that report increased complications with steeper angulations, as these may place more stress on the implant-bone interface and the prosthetic components. For example, a study found that steeper angulations (above 15°) increased the likelihood of screw loosening and fractures, which may be due to the increased mechanical forces acting on the components at higher angulations [15]. Hotinski *et al.* observed that implants designed to correct angulation demonstrated greater resistance to screw loosening when compared to straight implants [16]. From a clinical perspective, the results suggest that while 15° angulations may be clinically acceptable, 25° angulations lead to higher complication rates, which might influence the decision-making process when selecting abutments for anterior maxillary implants. The significant difference in screw loosening (p = 0.035) emphasizes the need for careful selection of abutment angulation to minimize long-term complications.

## Esthetic rating:

The esthetic outcomes were significantly better for layered zirconia compared to both monolithic zirconia and lithium disilicate (p = 0.004), with the highest Visual Analog Scale (VAS) scores observed for the layered zirconia at 0° angulation. The esthetic superiority of layered zirconia has been well-documented in the literature. According to Yousry *et al.* (2024), layered zirconia provides better translucency and mimics natural tooth structure more closely than monolithic zirconia, especially in anterior regions where esthetics are paramount [17]. Additionally, the VAS scores for the 0° abutment group were consistently higher than the 15° and 25° groups. This aligns with clinical experience, where a more straightforward implant placement (0° abutment angulation) allows for better alignment and placement of prostheses, leading to enhanced esthetic outcomes. Studies such as those by Perez *et al.* (2020) have shown that proper alignment of abutments correlates with higher esthetic satisfaction. The statistically significant difference in esthetic ratings between the 0° and 25° angulated groups further underscores the importance of precise angulation in achieving superior esthetic results [18].

## Patient satisfaction:

Patient satisfaction scores showed a clear trend, with the 0° abutment angulation group achieving the highest overall satisfaction (95%) compared to the 15° (90%) and 25° (85%) groups (p = 0.012). These findings highlight the importance of abutment angulation in patient comfort and overall satisfaction, which is supported by previous literature. According to a study by Lee *et al.* (2021), patient comfort and satisfaction are directly impacted by the positioning of the implant and the ability to provide a well-fitted prosthesis [19, 20]. The 0° angulation group likely benefitted from a more straightforward alignment and less occlusal interference, leading to greater comfort and functional satisfaction. Furthermore, comfort and esthetic satisfaction were rated significantly higher for the 0° group. This is clinically significant, as higher comfort levels and esthetic satisfaction can translate to improved patient outcomes and greater acceptance of the treatment. It also supports the clinical approach of using a more straightforward angulation whenever possible, as this not only reduces complications but also enhances patient-reported outcomes.

## Occlusal scheme:

The analysis of occlusal schemes showed that the canine-guided occlusion group had higher patient satisfaction scores across all domains-comfort, esthetics and function-compared to the group function occlusion group (p = 0.045). Canine-guided occlusion, which relies on the anterior teeth (especially the canines) to guide mandibular movement, has been shown to provide better functional outcomes and reduce stress on the implants [21]. This was confirmed by a study by Yesilyurt *et al.* (2021) [22], who found that canine-guided occlusion reduces lateral forces on the posterior teeth, thereby minimizing the risk of implant complications and improving overall patient satisfaction. The statistically significant difference (p = 0.045) in satisfaction between canine-guided and group function occlusion highlights the importance of occlusal scheme selection in implant rehabilitation. Clinically, it suggests that canine-guided occlusion should be prioritized, particularly for patients with implants in the anterior region, to improve long-term functionality and comfort.

## Clinical implications:

This study provides valuable insights into the clinical decision-making process when performing anterior maxillary implant placements with varying abutment angulations. The findings suggest that:

[1] Abutment angulation: A 0° abutment angulation should be preferred when possible to minimize complications such as screw loosening and fractures. Higher angulations (15° and 25°) are associated with increased complication rates and lower esthetic outcomes.

[2] Material selection: Layered zirconia outperforms both monolithic zirconia and lithium disilicate in terms of esthetic outcomes, making it the material of choice for anterior implants, especially when high esthetic expectations are required.

[3] Patient satisfaction: The 0° angulation and canine-guided occlusion combination leads to the highest patient satisfaction, indicating that implant placement should aim for the most straightforward alignment with optimal occlusal support.

These findings contribute to the growing body of literature that emphasizes the importance of careful planning and material selection in achieving optimal outcomes in implantology. Future studies with larger sample sizes and long-term follow-ups are needed to further validate these findings and explore the long-term impacts of abutment angulation and occlusal scheme on implant success and patient satisfaction.

## Limitation:

The study's limited sample size and single-center design may affect the generalizability of the results.

## Future perspective:

Further multicenter, longitudinal studies with larger sample sizes are needed to confirm these findings and explore long-term outcomes of different abutment angulations and materials.

## Conclusion:

Abutment angulation, material selection and occlusal scheme significantly influence the prosthetic success, esthetic outcomes and patient satisfaction in anterior maxillary implants. The 0° abutment angulation demonstrated the best clinical outcomes, with higher patient satisfaction and fewer complications. Layered zirconia was found to offer superior esthetic results. The canine-guided occlusion also contributed to better functional outcomes and patient comfort. These findings emphasize the importance of personalized treatment planning in optimizing implant prosthetic outcomes.
